# The Relationship of Obesity, Nutritional Status and Muscle Wasting in Patients Assessed for Liver Transplantation

**DOI:** 10.3390/nu11092097

**Published:** 2019-09-04

**Authors:** Helen Vidot, Katharine Kline, Robert Cheng, Liam Finegan, Amelia Lin, Elise Kempler, Simone I. Strasser, David Geoffrey Bowen, Geoffrey William McCaughan, Sharon Carey, Margaret Allman-Farinelli, Nicholas Adam Shackel

**Affiliations:** 1 Department Nutrition & Dietetics, Royal Prince Alfred Hospital, Camperdown, 2050 NSW, Australia; 2 Liver Injury and Cancer, Centenary Research Institute, The University of Sydney, Sydney, 2006 NSW, Australia; 3 Sydney Medical School, The University of Sydney, Sydney, 2006 NSW, Australia; 4 A.W. Morrow Gastroenterology and Liver Centre, Royal Prince Alfred Hospital, Camperdown, 2050 NSW, Australia; 5 School of Business, The University of Sydney, Sydney, 2006 NSW, Australia; 6 School of Life and Environmental Sciences Charles Perkins Centre, The University of Sydney, Sydney, 2006 NSW, Australia; 7 Medicine, University of New South Wales, Sydney, 2052 NSW, Australia

**Keywords:** obesity, muscle wasting, nutritional status, cirrhosis, liver transplantation

## Abstract

Introduction: Obesity co-exists with malnutrition and muscle atrophy in patients with cirrhosis. Muscle wasting is a feature of sarcopenia, a known determinant of patient outcomes. This is the first description of a relationship between obesity, subjective global assessment (SGA) of nutritional status and muscle wasting in patients with cirrhosis. Methods: The relationship between body mass index (BMI with obesity defined as ≥ 30 kg/m^2^), nutritional status (assessed by liver-specific subjective global assessment—SGA) and muscle wasting (assessed by corrected total cross-sectional psoas muscle area—cTPA) was analysed in patients with cirrhosis considered for liver transplantation between 1 January 2012 and 31 December 2014. Results: There were 205 patients, of whom 70% were males. The mean age was 52 ± 0.7 years and the Model for End-Stage Liver Disease (MELD) score was 16.8 ± 0.5. Overall, 31% of patients were obese and 56% of well-nourished (SGA A) individuals were obese. Muscle wasting was identified in 86% of all patients, irrespective of their nutritional status (A, B, C). All obese males classified as well-nourished (SGA A) were sarcopenic and 62% of obese females classified as SGA A were sarcopenic. Muscle wasting was worse in obese individuals (cTPA 230.9 mm^2^/m^2^ ± 12.9, *p* < 0.0001) and more likely to be associated with hepatic encephalopathy (*p* = 0.03). Univariate and multivariate analysis demonstrated testosterone deficiency was significantly associated with muscle wasting (*p* = 0.007) but not obesity (*p* = 0.8). Conclusion: Obesity combined with muscle wasting is common in patients with cirrhosis. Muscle wasting is common in well-nourished (SGA A) obese patients. Consequently, all patients assessed for liver transplantation should undergo additional screening for malnutrition and muscle wasting irrespective of BMI.

## 1. Introduction 

Malnutrition in chronic liver disease (CLD) is associated with poor quality of life and increased mortality and morbidity [1]. The underlying mechanisms of malnutrition in patients with cirrhosis are multifactorial and associated with abnormal macronutrient metabolism, malabsorption and decreased energy intakes due to disease-specific anorexia and early satiety [2,3,4]. The reported prevalence varies from 65% to 90% and is dependent on methods used to determine nutritional status [1,4]. The development of accurate assessment methods is important in both the assessment and management of malnutrition in patients with cirrhosis and in assessing the response to nutrition interventions [3,5].

Current methods of nutritional assessment include liver-specific subjective global assessment (SGA) [6], hand grip strength, anthropometric measurements of mid-arm circumference (MAC), triceps skinfold (TSF) and mid-arm muscle circumference (MAMC) [7,8]. Biochemical markers and BMI, per se, are poor indicators of nutritional status in patients with cirrhosis [7,9]. The increasing prevalence of obesity in patients with cirrhosis further complicates accurate nutritional assessment [10,11]. SGA is a validated tool for assessing nutritional status and has been described as the “gold standard” bedside assessment tool. It includes a physical examination of adiposity, muscle mass and oedema, and an assessment of functional capacity [12,13]. When combined, these factors are predictive of nutrition-associated complications [12,13,14]. The liver-specific SGA, which is widely used to assess the nutritional status of patients awaiting liver transplantation, includes additional components of disease severity [6]. It is a subjective composite score which incorporates a physical assessment of muscle wasting and subcutaneous adiposity in addition to gastrointestinal symptoms, functional capacity and components of disease severity, such as the presence or absence of varices, hepatic encephalopathy, recurrent infection and renal compromise [6].

Obesity is associated with an increased risk of developing chronic disease [11,15] and non-alcoholic liver disease (NAFLD) is increasingly recognised as a primary factor in the development and progression of hepatic cirrhosis [3]. Obese patients with chronic liver disease have a higher rate of progression to clinical decompensation than patients within the healthy weight range [10]. The Australian Bureau of Statistics projects a 2.5-fold increase in obesity within the next 20 years [16], which has significant clinical implications, including an increased prevalence of NAFLD which may previously have been under-recognised [17].

Muscle atrophy is a feature of sarcopenia. Sarcopenia is defined as both a loss of skeletal muscle mass [18] and a loss of muscle strength [18,19,20]. It is frequently associated with aging, chronic diseases and malignancies [2,21,22]. Although more often associated with aging, sarcopenia may present in people of any age [20]. Malnutrition, protein malnutrition in particular [18], malabsorption and metabolic abnormalities contribute to the development of sarcopenia in patients with CLD [23]. A recent study in patients with cirrhosis reported poor outcomes in sarcopenic obese patients [11].

A recent expert opinion highlights the importance of sarcopenia in liver transplantation and recommends standardised assessment [24]. Sarcopenia has been reported in up to 70% of patients with CLD [24,25] and in 22%–70% of patients on the liver transplant waitlist [26]. Sarcopenic obesity is present in 20%–40% of liver transplant candidates and is strongly associated with non-alcoholic fatty liver disease (NAFLD) as the primary cause of cirrhosis [15]. It is unclear if the mix of aetiologies determines the prevalence of the problem and if this remains a problem when NAFLD is not a dominant indication for transplant [27]. 

The European Association for the Study of the Liver (EASL) clinical practice guidelines for the nutritional management of chronic liver disease define sarcopenia as loss of muscle mass with consequent loss of strength [3]. The reported prevalence is dependent on the sample population, method of assessment and definition of sarcopenia [28,29]. Techniques used to assess sarcopenia and muscle atrophy include anthropometry, hand grip strength, walking speed, DXA and computed tomography (CT) imaging [14,28,30] CT imaging at the third lumbar vertebra is the preferred technique for assessing sarcopenia in cirrhotic patients [18,28,30].

We aimed to explore the relationship between obesity, liver-specific SGA, and muscle wasting as a feature of sarcopenia, in addition to the symptoms of portal hypertension, ascites and hepatic encephalopathy, in a group of patients with mixed disease aetiologies undergoing assessment for liver transplantation. 

In addition, as CT imaging is costly and unable to be performed routinely, we aimed to develop an alternative measure of sarcopenia, which is easier and less costly to assess, by examining two additional muscle groups (either anterior or lateral abdominal wall instead of psoas muscle).

## 2. Methods

### 2.1. Ethics Consideration

Approval to access health data and medical records was granted by the Sydney Local Health District, Royal Prince Alfred Hospital (RPAH) Ethics Review Committee: HREC/15/RPAH/275. The ethics protocol was considered to be a low- or negligible-risk study with a waiver of consent. 

### 2.2. Patient Cohort

**Figure 1:**
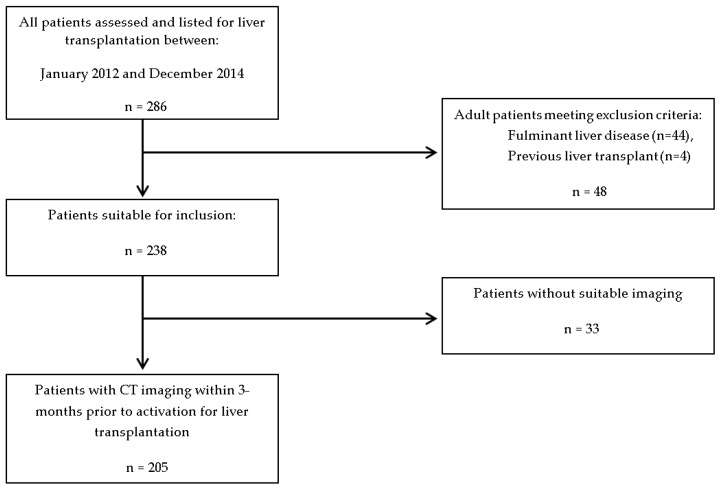
Patient selection. Study design and selection criteria of patients assessed for liver transplantation from 2012 to 2014.

This report is a retrospective investigation of all adult patients assessed and activated on the waiting list for transplantation between 1 January 2012 and 31 December 2014 at a single quaternary hospital. Patients who presented with fulminant disease were excluded due to the nature of their presentation. Patients who were listed for a second or third transplant and patients who did not have adequate computer tomography (CT) imaging were excluded from further analysis (Figure 1).

### 2.3. Assessment 

Patient assessment for liver transplantation was performed by clinical staff. All patients with CLD referred to the liver transplant clinic at this centre are assessed. Patients not suitable were excluded from the analysis, including those who had ongoing illicit drug use, current alcohol use and unfavourable social supports. Demographic information, indication for liver transplantation, and primary and secondary diagnoses were obtained from the database. Biochemical markers were collated from blood results at the time of assessment and included bilirubin, albumin, creatinine, testosterone, international normalized ratio (INR), alkaline phosphatase (ALP), gamma glutamyl transferase (GGT), alanine aminotransferase (ALT) and aspartate aminotransferase (AST). Waiting list times and waitlist mortality data was also collected from the database. 

For the purposes of this investigation, muscle wasting was defined by the cross-sectional area of the psoas muscle at the third lumbar vertebra (L-3), adjusted for height. The cTPA index has been recognised as an objective measure of muscle wasting in cirrhosis [28]. The formula for the calculation of the corrected total psoas muscle area (cTPA) is [31]:

$$\left\{cTPA=\left[AP\;psoas\;length\left(mm\right)\times Transverse\;psoas\;length\left(mm\right)\right]÷height\;{\left(m\right)}^{2}\right\}$$

**Figure 2:**
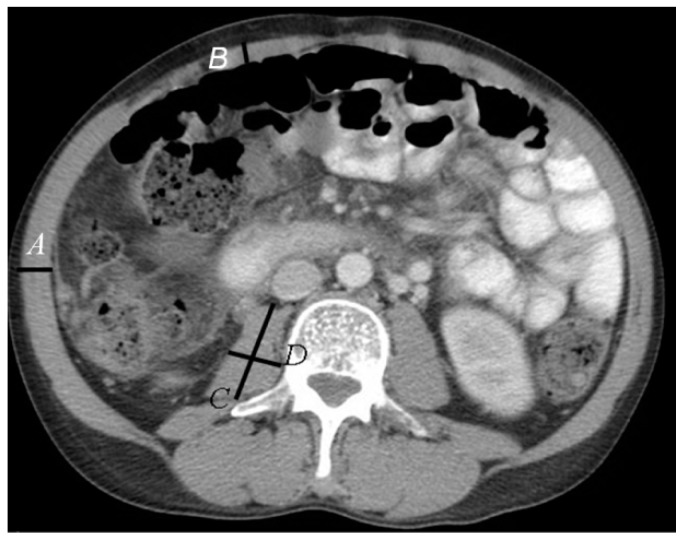
Assessment of muscle wasting at the third lumbar vertebra (L-3). Cross-sectional psoas muscle area measured using computed tomography imaging (CT) at the level of the third lumbar vertebra: **A:** lateral wall muscle measurement; **B:** abdominal wall muscle 2 cm to the right of the midline; **C:** anterior/posterior length measurement; **D:** transverse psoas diameter.

CT imaging performed within 3 months of transplant activation was used to determine psoas muscle area. All CT images were viewed on an imaging station by two independent blinded observers (AL and KK). Gender-specific cut off points for muscle wasting were 545 mm^2^/m^2^ for males and 385 mm^2^/m^2^ for females [31]. Additional measurements included abdominal wall muscle 2 cm to the right of the midline at L3 and lateral wall muscle at the most lateral point of the abdomen at L3 (Figure 2).

Clinical assessment of patients’ physical and cognitive state was determined by a physician at the time of assessment and the West Haven Criteria used to identify hepatic encephalopathy (HE) [32]. Child Turcotte Pugh (CTP) [33] and the Model for End-Stage Liver Disease (MELD) scores [34] were calculated to indicate disease severity. Disease aetiology and the presence of ascites and hepatocellular carcinoma (HCC) were recorded by the treating physician using current guidelines at the time of assessment [35,36,37]. 

Liver-specific SGA [6] was performed within 3 months of CT imaging by 2 specialist clinical dietitians, each with more than 10 years of clinical experience in this area. Body mass index (BMI) at the time of nutritional assessment was adjusted for ascites using the method described by Madden and Wicks [38]. Obesity was defined as BMI ≥ 30 kg/m^2^ [39].

Only patients for whom all clinical variables were available were included in the analysis. Results are reported as percentages of the entire cohort. Categorical variables were analysed using Chi Squared ($\chi^{2}$), and continuous results were compared using the Student’s T-test. Multiple comparisons were made using one-way analysis of variance (ANOVA) followed by Tukey’s multiple comparison or the Kruskal–Wallis test for non-normally distributed data. Relationships between quantitative variables were assessed by Pearson correlation analysis. The threshold for statistical significance was *p* < 0.05 and all tests were two-tailed. SAS (Statistical Analysis System) was used for multivariate analysis of variables contributing to the development of sarcopenia. 

The relationship between ascites, obesity, and SGA classification, in addition to the relationship between hepatic encephalopathy, obesity and SGA classifications, were further examined using logit regression [40]. 

## 3. Results

### 3.1. Demographics

Two hundred and eighty-six patients were identified from the assessment database of the Liver Transplant Unit at RPAH. Two hundred and five patients with a mean age of 52.8 ± 0.7 years were included in the analytic sample (Figure 2) due to the availability of a complete data set. Adult patients with fulminant disease (*n* = 44), those listed for re-transplantation (*n* = 4) and those with incomplete CT imaging (*n* = 33) were excluded from the analysis. The demographics of the group are outlined in Table 1. 

Viral hepatitis, including hepatitis B and hepatitis C, was the most common primary aetiology of cirrhosis, followed by cholestatic liver disease, alcoholic liver disease and non-alcoholic fatty liver disease (NAFLD)/non-alcoholic steatohepatitis (NASH). NAFLD was seen as the primary indication for transplant in only 7% of individuals.

Eighty-six percent of the entire cohort patients had significant muscle wasting. Males had a lower mean cTPA (267.8 mm^2^/m^2^ ± 95.8) than females (381.1 mm^2^/m^2^ ± 90.3) (*p* < 0.001), and 97% of males and 63% of females were sarcopenic. Age and BMI did not differ significantly between the muscle-wasted and non-muscle-wasted groups. There was a higher prevalence of muscle wasting in patients with a primary diagnosis of NAFLD/NASH (*p* = 0.009). 

### 3.2. Obesity, SGA and Sarcopenia, 

The relationship between obesity, nutritional status, muscle wasting and gender is outlined in Table 2. Obesity was identified in 31% of all patients (BMI ≥ 30). Eighty-nine percent of obese patients and 86% of non-obese patients had significant muscle wasting had. Obese patients had the lowest cTPA measurements (230.9 mm^2^/m^2^ ± 12.9) (*p* < 0.0001). 

**Figure 3:**
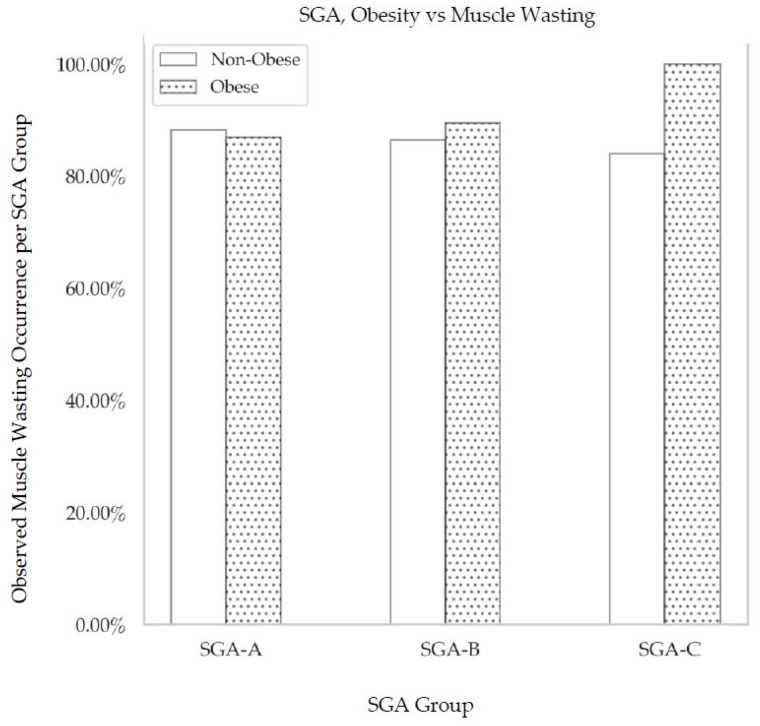
The distribution of sarcopenia across SGA categories. SGA, subjective assessment of nutritional status: SGA A, well nourished; SGA B, moderately malnourished; SGA C, severely malnourished. The observed percentage of muscle wasting occurrence per SGA group did not vary significantly across SGA groups based on either obesity or nutritional status.

The observed percentage of muscle wasting per SGA group is shown in Figure 3.

**Figure 4:**
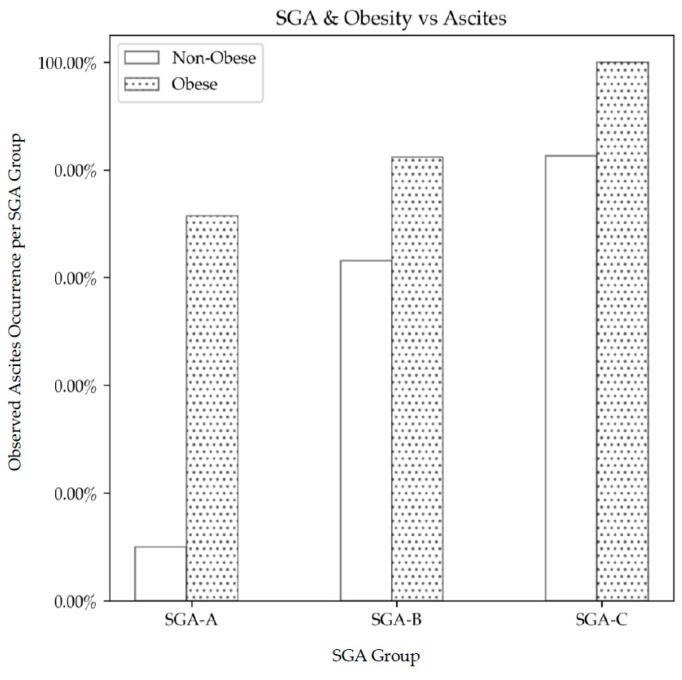
The distribution of ascites and obesity across SGA categories. There was a significant increase in ascites in obese well-nourished (SGA A) patients compared to non-obese well-nourished patients (*p* = 0.007).

As predicted, obesity was more common in well-nourished (SGA A) patients (86%) than in moderately malnourished (SGA B) patients (67%) or severely malnourished (SGA C) patients (7%). SGA B patients were more likely to have ascites compared to SGA A (*p* = 0.002) (Figure 4). Similarly, SGA C patients were more likely to have ascites when compared to SGA A (*p* = 0.012). The presence of the significant complications of portal hypertension with ascites and/or hepatic encephalopathy was not significantly different in obese individuals, irrespective of nutritional status. In obese patients, the percentage occurrence per SGA group was SGA A 86%, SGA B 76% and SGA C 93%. 

All males with a BMI ≥ 30 kg/m^2^ who were classified as well-nourished (SGA A) had significant muscle wasting. The prevalence of muscle wasting in moderately malnourished (SGA B), obese individuals was 89% and 100% in obese, severely malnourished (SGA C) individuals. The prevalence of muscle wasting in non-obese patients who were well nourished was 88%. 

The observed increase in the percentage of ascites occurrence in the obese moderately malnourished group compared to the non-obese moderately malnourished group did not reach statistical significance.

**Figure 5:**
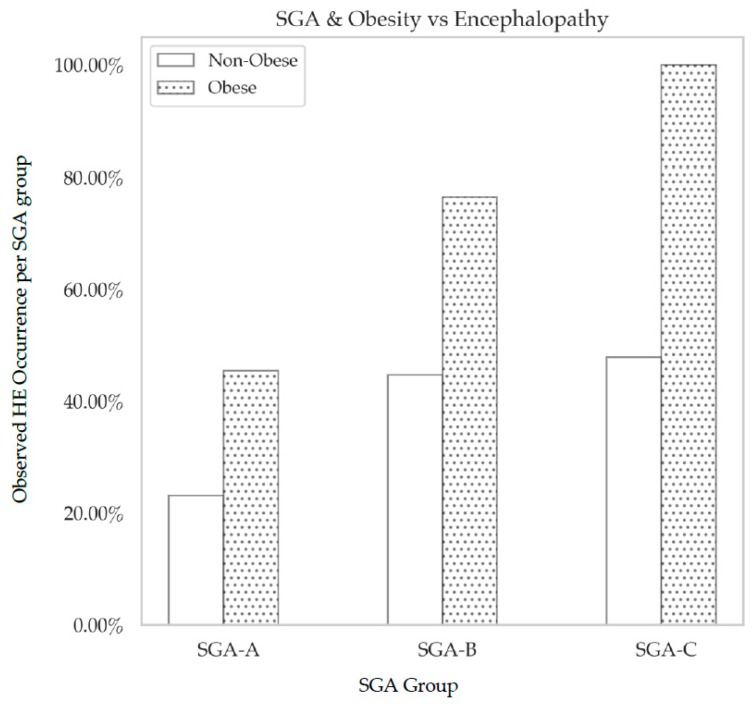
The distribution of HE and obesity across SGA categories. There was an observed trend towards an increased occurrence of HE as a function of muscle wasting, as well as a function of obesity. There was a significant increase in the observed percentage of HE occurrence in obese, moderately malnourished (SGA B) patients compared to well-nourished (SGA A) patients (*p* = 0.03).

Logit regression was used to examine the effect of obesity with SGA and HE. There was a significant increase in HE in obese, moderately malnourished patients compared to the non-obese moderately malnourished group (*p* = 0.027) (Figure 5).

### 3.3. Clinical Significance of Muscle Wasting

Patients with significant muscle wasting were more likely to be clinically decompensated (CTP B or C) (*p* = 0.03) but there was no difference between the muscle-wasted and non-muscle-wasted groups with respect to MELD scores < 30 (*p* = 0.02). Creatinine concentrations in patients who were sarcopenic were higher than in those who were not sarcopenic (84.5 µmol/L, 71.6 µmol/L, respectively) (*p* = 0.05). Univariate and multivariate analysis of the stratified MELD scores indicated that muscle wasting was associated with increased disease severity (MELD between 20–30) (*p* = 0.005) (Table 3). 

The prevalence of muscle wasting was higher in patients with HE (*p* = 0.03). There was a trend towards an increased prevalence of HE in obese patients with increasing degrees of sarcopenia (*p* = 0.06). Psoas muscle depletion was greater in males who were encephalopathic (211.0 ± 4.7 mm^2^/m^2^) than males who were not encephalopathic (309.6 ± 4.7 mm^2^/m^2^) (*p* < 0.0001) and similarly, in females who were encephalopathic (276.8 ± 38.7 mm^2^/m^2^) compared to females who were not encephalopathic HE (355.1 ± 14.1 mm^2^/m^2^) (*p* = 0.02). Males had a significantly lower cTPA (*p* < 0.0001).

### 3.4. cTPA-Defined Muscle Wasting and other Muscle Groups

There was a significant correlation between cTPA and median anterior abdominal muscle thickness (Pearson *r* = 0.31, *p* < 0.0001) and cTPA and median lateral abdominal muscle thickness (Pearson *r* = 0.46, *p* < 0.0001). Anterior abdominal muscle thickness was lower in males (*p* = 0.006). 

There was a significant correlation between anterior abdominal muscle and lateral abdominal muscle thickness (Pearson *r* = 0.26, *p* < 0.001). One-way ANOVA analysis comparing cTPA with obesity confirmed a significant difference in median lateral muscle wall thicknesses between sarcopenic and non-sarcopenic patients (*p* = 0.02). Obese patients with psoas muscle wasting had the lowest median anterior abdominal muscle thickness (*p* = 0.004) and the lowest median lateral abdominal muscle thickness (*p* = 0.003). 

### 3.5. Serum Testosterone Concentrations and Muscle Wasting 

Testosterone concentrations were lower in both males and females with psoas muscle wasting compared to those who did not have psoas muscle wasting [12.1 nmol/L, 13.7 nmol/L, respectively] (*p* < 0.0001). All males had testosterone concentrations below the normal range, irrespective of muscle wasting or BMI [muscle wasting, BMI ≥ 30, 12.5 ± 10.5 nmol/L; muscle wasting, BMI < 30, 14.9 ± 9.7 nmol/L; no muscle wasting, 13.7 ± 4.8 nmol/L] (*p* = 0.41). There was a significant inverse correlation between testosterone and both cTPA and anterior abdominal muscle thickness for males and females (r = −0.29, *p* = 0.001 and r = −0.27, *p* = 0.003, respectively).

Table 3 shows the univariate logistic regression analysis for the independent variables suggested to be associated with muscle wasting. When all variables were modelled, only testosterone remained significantly associated with muscle wasting (OR 1.72; CI 1.16–2.56; *p* = 0.007). SGA was not included as it is recognised to be dependent on the other variables. 

### 3.6. Survival on the Waiting List 

There was no difference in waitlist mortality between muscle-wasted and non-muscle-wasted patients (*p* = 0.31). The mean waitlist time was 214 ± 29 days for patients with muscle wasting and 271 ± 97 days for patients who were not muscle wasted (*p* = 0.38). The overall survival of the group was 72%, with 69% proceeding to liver transplantation. Sixteen percent of patients died prior to transplantation or were permanently removed from the transplant waitlist. At the time of this analysis, 13% of patients were waiting for transplantation. 

## 4. Discussion

Muscle wasting, in combination with obesity, was found to be a common occurrence in patients with cirrhosis assessed for liver transplant during this period and was irrespective of the aetiology of liver failure. This significantly advances our understanding of the problem of sarcopenic obesity as it occurs in the majority of obese patients, not just those with NAFLD as a primary diagnosis.

This high prevalence of muscle wasting in patients on the liver transplant waiting list is consistent with other studies [41,42]. Obesity was found to be associated with a higher level of muscle wasting and the high prevalence of muscle wasting, ascites and HE in obese, well-nourished (SGA A) individuals was an unexpected finding.

Malnutrition in patients with cirrhosis has been shown to correlate with disease severity [3,14]. These results are consistent with previous reports that deteriorating nutritional status is associated with an increase in both the symptoms of advanced liver disease and muscle atrophy [14]. The prevalence of muscle wasting and the symptoms of portal hypertension in obese, well-nourished patients is concerning. These individuals are the least likely to receive nutrition intervention due to the lack of recognition of nutritional compromise and muscle wasting using the liver-specific SGA alone. 

The combination of obesity and muscle wasting is associated with increased frailty, disability and quality of life and does not protect against chronic disease-related mortality and morbidity [43]. Historically, medical professionals have been reported to systematically avoid the “problem” of obesity and consider their involvement to be futile [44]. Recent evidence indicates that the majority of physicians continue to regard obesity as a chronic, behavioural condition and consider themselves ill-equipped to treat obesity [45]. However, early recognition and management at the primary care level is important and has the potential to improve disease outcomes [45]. 

Body composition and muscle mass have been identified as surrogate markers for sarcopenia [15]. The current study demonstrates that the liver-specific SGA alone does not detect muscle wasting, particularly in the presence of obesity. Well-nourished, obese males with the highest degree of muscle wasting were least likely to be classified as malnourished. Previous reports of patients with respiratory failure have demonstrated that SGA failed to identify muscle wasting in 60% of patients classified as well-nourished (SGA A) [46]. 

Our results imply that the liver-specific SGA may be adequate to assess muscle wasting in non-obese patients who are malnourished. However, it is important that both obese and non-obese patients assessed for liver transplantation routinely undergo a more detailed investigation of their muscle mass as part of their nutritional assessment. Alternative assessments would include an assessment of hand grip strength, anthropometric measurements of mid-arm circumference, midarm triceps skinfold and mid-arm muscle circumference, thigh muscle ultrasound measurements combined with an assessment of functionality (six-minute walk test, gait speed, Get Up and Go test, and stair climb power) [47,48]. These measures could also be used to assess response to nutrition intervention [48]. 

There is increasing recognition that the pathogenesis of both covert and overt HE in patients with cirrhosis is linked with increasing concentrations of ammonia and decreased muscle mass [49,50,51]. Our results confirmed an association between HE and muscle wasting. It is also important to recognise the role of obesity in the development of the symptoms of clinical decompensation. Obese, well-nourished patients with a higher prevalence of muscle wasting had a higher prevalence of HE than their non-obese counterparts. 

Muscle atrophy was identified more frequently in males than females which is also consistent with previous observations and reflects the gender distribution of both CLD and sarcopenia [14,52]. Sarcopenia in obese patients is associated with poorer outcomes and survival [11,47] and many studies report an increased waitlist mortality in sarcopenic patients with increased complications [41,42,53]. Obese sarcopenic patients with NASH are reported to have higher MELD scores and longer wait-list times [3]. Our results did not demonstrate a significant difference in the waiting list times between patients with or without muscle depletion, including obese and non-obese individuals.

Testosterone both stimulates and reduces muscle protein synthesis [54]. Testosterone targets androgen receptors in muscle cells to promote growth and triggers myocyte formation in satellite cells [54]. It also acts to reduce muscle protein degradation in males by down-regulating myostatin and upregulating insulin-like growth factor-1 [54]. Low serum testosterone concentrations contribute to muscle wasting, frailty and mortality [54,55,56] and have been identified in up to 90% of males with cirrhosis [54]. In addition, population studies have demonstrated an increased prevalence of testosterone deficiency in obese males [57,58]. In the current study, testosterone concentrations were low in all male patients, irrespective of the presence of muscle wasting and/or obesity. Testosterone was found to be independently associated with muscle wasting in both males and females. Disease severity alone was not an independent predictor in the development of muscle wasting in this group but, interestingly, a higher MELD score was associated with muscle wasting. This would suggest that muscle wasting and low testosterone concentrations are indicators of worsening liver disease. 

A novel aspect of the current study was measurement of the thickness of both the anterior abdominal wall muscle and the lateral abdominal wall muscle at the L3 vertebral level. These muscles are potentially easier to identify and measure than the psoas in a clinical setting, possibly using modalities such as ultrasound. However, further investigation of these results is required to confirm these findings. 

At the time this study was designed, the cTPA cut-off values for the diagnosis of sarcopenia were those identified by Jones et al. [31]. Recent reports of alternative cTPA values for male patients with end-stage liver disease waiting for liver transplantation [59] were derived by evaluation of the relationship between cTPA and waitlist mortality, which has multiple determinants in addition to sarcopenia.

An expected consequence of the retrospective nature of this study is the incompleteness of the data. However, the results reflect real-world data and include variations in adherence to protocols as occurs in clinical practice. Hand grip strength, a reliable indicator of muscle strength in CLD [9], was measured in a small number of subjects due to the part-time involvement of the dietitians at the time of this study and was not reported. Future prospective investigations should include a more comprehensive assessment of sarcopenia using more specific assessment tools, such as those previously discussed [44,45].

Accurate assessment of sarcopenia would assist in targeted nutrition intervention therapies. These would include strategies to ensure patients meet the recommended protein intake of 1.2–1.5 g/kg/day, reducing fasting periods to minimise hepatic glycogen depletion and branched-chain amino acid supplementation, in addition to endurance and resistance exercises, hormone replacement therapy and ammonia-lowering strategies [3,20].

## 5. Conclusions

Muscle wasting, in combination with obesity, is common in patients with end-stage cirrhosis awaiting transplant, irrespective of disease aetiology. Liver-specific SGA alone is a measure of nutritional status rather than body composition and does not identify muscle atrophy in well-nourished, obese patients undergoing assessment for liver transplantation. A comprehensive assessment of body composition, sarcopenia, ascites and HE should be included routinely by clinicians as part of the evaluation of nutritional status in all patients with cirrhosis. 

## Figures and Tables

**Table 1:** Cohort Demographics.

	All	Muscle Wasting	No Muscle Wasting
Gender			
Males	143	139	**4** *
Females	62	39	23
Age-years (mean±SEM)	52.8 ± 0.7	52.9 ± 1.0	52.0 ± 2.0
CTP: (% A/B/C)	17/38/45	17/31/41	**0/7/4** *
MELD (mean ± SEM)	16.8 ± 0.5	17.7 ± 1.0	17.9 ± 0.5
HCC			
Primary diagnosis (%)	2	2	0
Secondary diagnosis (%)	20	19	1
Aetiology			
Viral (%)	48	42	6
EtOH (%)	14	12	1
NASH (%)	7	6	1
Cholestatic (%)	15	12	3
Other (%)	12	10	2
SGA			
A (well-nourished) (%)	41	**36** **	5
B (moderately malnourished) (%)	79	**68** **	11
C (severely malnourished) (%)	30	**26** **	4
HE			
None (%)	45	39	**3** *
Grade 1–2 (%)	39	36	6
BMI (mean ± SEM)	27.9 ± 0.4	27.7±0.4	27.6 ± 1.1
Corrected psoas muscle area (mm^2^/m^2^) (mean ± SEM)	301.1 ± 7.5	**273.4 ± 6.0** *	**483.8 ± 15.5** *

CTP, Child Turcotte Pugh; MELD, Model for end-stage liver disease; HCC, hepatocellular carcinoma; EtOH, alcohol; NASH, non-alcoholic steatohepatitis; SGA, subjective global assessment of nutritional status (liver specific); HE, hepatic encephalopathy; BMI, body mass index. * significant difference (*p* < 0.05) between muscle-wasted and non-muscle-wasted groups. ** significant difference (*p* < 0.05) between well-nourished (SGA A) and malnourished (SGA B and C) groups. Bolded to indicate significance.

**Table 2:** Body mass index (BMI), subjective global assessment of nutritional status (SGA) and muscle wasting.

% Patients with Significant Muscle Wasting per SGA Group			
Participant Groups (% of Group)	SGA A	SGA B	SGA C
All Patients (86%)			
Males	17	36	12
Females	7	9	5
Obese Patients (89%)			
Males	34	34	5
Females	11	5	0
Non-obese Patients (86%)			
Males	10	39	14
Females	4	12	7

Obese: BMI ≥ 30 kg/m^2^; non-obese: BMI < 30 kg/m^2^; SGA A, well-nourished; SGA B, moderately malnourished; SGA C, severely malnourished. There were no significant differences between BMI, gender and obesity using Kappa measure of difference analysis.

**Table 3:** Univariate logistic regression analyses of variables as potential predictors of sarcopenia in patients on the liver transplant waiting list.

Variable (*n* = 205)	Mean		Univariate	
	Muscle Wasting	No Muscle Wasting	OR (95% CI)	*p*-Value
Gender				
Male (n)	139	4	20.5 (6.7–62.8)	<0.0001
Female (n)	39	23		
Age (SEM)	52.9 ± 0.8	52 ± 1.7	1.0 (0.97–1.05)	0.68
MELD	16.3 ± 6.0	16.4 ± 5.9	0.97 (0.91–1.03)	0.32
CTP (%A/ B/ C)	17/31/41	0/7/4	0.91 (0.724–1.134)	0.39
Albumin (SEM) (g/L)	32.3 ± 0.5	33.2 ± 1.4	0.98(0.916–1.044)	0.50
BMI (kg/m^2^) (SEM)	28.0 ± 0.4	27.6 ± 1.1	1.01 (0.928–1.101)	0.80
Psoas muscle area (mm^2^/m^2^) (SEM)	275 ± 6.0	484 ± 15.5	0.97 (0.59–0.980)	<0.0001
Anterior abdominal muscle (mm) (SEM)	6.0 ± 0.2	6.9 ± 0.6	0.90 (0.796–1.03)	0.12
Lateral abdominal muscle (mm) (SEM)	15.2 ± 0.4	17.6 ± 0.7	0.91 (0.84–0.98)	0. 02
Testosterone (SEM) (nmol/L)	12.1 ± 1.0	13.7 ± 1.3	1.2 (1.07–1.40)	0.004
ALT (U/L)	81 ± 13	77 ± 11	0.99 (0.998–1.00)	0.29

CTP, Child Turcotte Pugh; MELD, model for end-stage liver disease; HCC, hepatocellular carcinoma; EtOH, alcohol; NASH, non-alcoholic steatohepatitis; SGA, subjective global assessment of nutritional status (liver specific); HE, hepatic encephalopathy; BMI, body mass index.
